# A Biomimetic 3D‐Self‐Forming Approach for Microvascular Scaffolds

**DOI:** 10.1002/advs.201903553

**Published:** 2020-03-01

**Authors:** Liucheng Zhang, Yi Xiang, Hongbo Zhang, Liying Cheng, Xiyuan Mao, Ning An, Lu Zhang, Jinxiong Zhou, Lianfu Deng, Yuguang Zhang, Xiaoming Sun, Hélder A. Santos, Wenguo Cui

**Affiliations:** ^1^ Department of Plastic and Reconstructive Surgery Shanghai Ninth People's Hospital Shanghai JiaoTong University School of Medicine 639 Zhi Zao Ju Road Shanghai 200011 P. R. China; ^2^ Shanghai Key Laboratory for Prevention and Treatment of Bone and Joint Diseases Shanghai Institute of Traumatology and Orthopaedics Ruijin Hospital Shanghai Jiao Tong University School of Medicine 197 Ruijin 2nd Road Shanghai 200025 P. R. China; ^3^ Department of Pharmaceutical Sciences Laboratory and Turku Center for Biotechnology Åbo Akademi University Turku FI‐20520 Finland; ^4^ State Key Laboratory for Strength and Vibration of Mechanical Structures School of Aerospace Xi'an Jiaotong University Xi'an 710049 P. R. China; ^5^ Drug Research Program Division of Pharmaceutical Chemistry and Technology Faculty of Pharmacy University of Helsinki Helsinki FI‐00014 Finland; ^6^ Helsinki Institute of Life Science (HiLIFE) University of Helsinki Helsinki FI‐00014 Finland

**Keywords:** bioinspired materials, biomimetic microvessels, self‐forming, vascular scaffolds

## Abstract

The development of science and technology often drew lessons from natural phenomena. Herein, inspired by drying‐driven curling of apple peels, hydrogel‐based micro‐scaled hollow tubules (MHTs) are proposed for biomimicking microvessels, which promote microcirculation and improve the survival of random skin flaps. MHTs with various pipeline structures are fabricated using hydrogel in corresponding shapes, such as Y‐branches, anastomosis rings, and triangle loops. Adjustable diameters can be achieved by altering the concentration and cross‐linking time of the hydrogel. Based on this rationale, biomimetic microvessels with diameters of 50–500 µm are cultivated in vitro by coculture of MHTs and human umbilical vein endothelial cells. In vivo studies show their excellent performance to promote microcirculation and improve the survival of random skin flaps. In conclusion, the present work proposes and validifies a biomimetic 3D self‐forming method for the fabrication of biomimetic vessels and microvascular scaffolds with high biocompatibility and stability based on hydrogel materials, such as gelatin and hyaluronic acid.

## Graphical abstract

Inspired by drying‐driven curling of apple peels, hydrogel‐based micro‐scaled hollow tubules are proposed for biomimicking microvessels with diameters of 50–500 µm, which promote microcirculation and improve the survival of random skin flaps. A 3D‐shape‐morphing technique is of great flexibility and potential to lay the foundation for the construction of complex vascular networks, such as Y‐branches, anastomosis rings, and triangle loops.

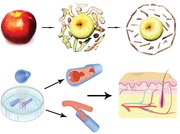

Recently, some materials became attractive for their self‐forming properties. Self‐forming properties of 2D materials can transform into complex 3D constructions, responding to their varying internal strains. For example, López‐Valdeolivas et al. reported secondary molding of liquid crystalline elastomer to form a 3D structure taking advantage of the thermosensitivity of the material.^[^ ^1^ ^]^ Kuribayashi‐Shigetomi et al. folded a planar material into a polyhedron via cellular traction.^[^ ^2^ ^]^ Moreover, hollow tubular structure was formed by stress‐induced rolling of electrospun PLGA/PCL mats, reported by Cheng et al.^[^ ^3^ ^]^ Thus, based on the self‐forming property, it is possible to propose a 3D self‐forming method to fabricate microvessel scaffolds with great significance.

Being the track in which nutrients and metabolites are continuously transported and circulated, the complex vascular networks are essential for the survival and repair of tissues.^[^ ^4^, ^5^ ^]^ Problems, such as flap necrosis,^[^ ^6^, ^7^ ^]^ coronary heart disease,^[^ ^8^, ^9^ ^]^ and bone necrosis,^[^ ^10^, ^11^ ^]^ often arise once the circulation is interrupted. To address these problems, many studies have been devoted to promote the progress of vascularization in which various angiogenic cells migrated to form primary microvessels, which further develop into arterioles and venules.^[^ ^5^, ^12^ ^]^ These studies were often conducted with therapies based on functional cells (e.g., adipose‐derived stem cells),^[^ ^13^ ^]^ cytokines (e.g., vascular endothelial growth factor),^[^ ^14^ ^]^ drugs (e.g., deferoxamine)^[^ ^15^ ^]^ to improve blood supply, accelerate tissue regeneration or reduce inflammation. However, satisfactory outcomes have rarely been achieved with the following problems in practice: 1) lack of normal tissue structure made it difficult for transplant cells to survive and differentiate; 2) the nonuniform distribution of cytokines tend to harm the normal tissue; 3) the release and clearance of drugs are unpredictable and unmanageable; and 4) the reconstruction of the microcirculation is time‐consuming for the microvessels to grow from neonatal to mature.^[^ ^5^, ^6^, ^10^, ^16^ ^]^ Overall, the nodus is to regulate the local micro‐vascularization rationally, directionally and rapidly. Herein, the in vitro construction of micro‐scaffold for vessels and microvessels with 3D‐biomimetic structure provides a new perspective for the practice of pre‐vascularization.

Currently, three major methods have been proposed for the construction of 3D hollow tubules: 1) subtractive manufacturing by carving to fabricate millimeter‐scaled vascular scaffolds;^[^ ^16^ ^]^ 2) precise knitting of fasciculus to construct micro‐scaled artificial tubules;^[^ ^17^ ^]^ and 3) additive manufacturing by 3D printing to print millimeter‐scaled tubules.^[^ ^18^ ^]^ However, owing to the limited accuracy of these methods, the fabrication of tubules smaller than 600 µm in diameter remains a challenge.^[^ ^19^ ^]^ In addition, hollow structures are not completely remodeled with these methods, and the complicated fabricating procedures are extremely difficult to control. For example, the simulation and remodeling of haversian canal in bones (φ = 60–90 µm),^[^ ^20^ ^]^ microvessels in flaps (φ = 20–500 µm),^[^ ^21^, ^22^, ^23^ ^]^ and retinal vessels (φ = 20–200 µm)^[^ ^24^, ^25^ ^]^ are still very difficult to be realized. Therefore, exploration on the early stage of microvessel regeneration is crucial and difficult. However, with the 3D self‐forming property, it is feasible to solve this problem.

Herein, inspired by the dried apple peels that spontaneously curl into hollow tubules, and tubules of various diameters and structures, we fabricated micro‐scaled hollow tubules (MHTs) via curling of planar materials, driven by the difference in contractility and swelling ratio between its upper and lower layers. Taking biocompatibility, cytotoxicity, and degradation properties into consideration, photocrosslinkable gelatin methacrylamide (GelMA) or methacrylated hyaluronic acid were used. Furthermore, since the “apple peels” with different shapes resulted in different tubulars, the diameter (50–500 µm) of the microvessel scaffold was regulated by adjusting the concentration and UV‐exposure time. The MHTs were demonstrated to be of good tensile strength and cytocompatibility. In the in vitro studies, the endothelial cells seeded on the MHTs grew and migrated normally, tight junctions and vessel walls was observed after 3D culture, which finally formed manufactured vessels as a microvessel scaffold. The scaffold was further implanted into random flap model in rats for in vivo validation. Compared to the control group, the microvessel scaffolds significantly reduced necrosis area and promoted the microvessel density (MVD) of the flaps. Overall, inspired by curling phenomenon of apple peels, we developed a facile method to fabricate biomimetic microvessel scaffold based on hydrogels with high accuracy, controllability, and handleability, which is a promising solution to the tough question of early forming of microvessel in the practice of tissue engineering.

Referring to the drying‐driven curling of “apple peels” (**Figure** **1**a), hydrogel flakes were first prepared. GelMA was chosen as the matrix material due to its good biocompatibility, mesoporous structure, and inexpensiveness.^[^ ^4^, ^6^ ^]^ Rectangular‐shaped GelMA hydrogel flake (approximately 20 μL GelMA) with the dimensions of 30 mm × 2 mm × 0.20 mm was fabricated by 3D printer (CELLINK AB, Sweden) or glass mould‐followed by immediate UV exposure for solidification. The flake was then dried by air‐blowing at room temperature (RT) or baking at 60 °C. At this point, it was observed that the GelMA flake was going through centripetal contraction as it was dehydrated, but the curling did not happen, because the flake was stuck to the slide. Therefore, the flake was soaked in water to re‐swell, and the flake curled into an MHT in the same way as “apple peels” did (Figure 1b). There are two explanation for this phenomenon: 1) during the photocrosslinking, the upper layer of the flake absorbed more UV energy, resulting in denser polymerization, thus the swelling ratio of the upper layer was lower than that of the lower layer (Figure S1, Supporting Information); 2) Since the drying happened sequentially, the upper layer of the flake contracted more strongly, driving the internal of the hydrogel to curl centripetally. Consequently, the hydrogel flake curled spontaneously as shown in Figure 1l,m, driven by the centripetal contraction force led by the upper layer and the higher swelling ratio of the lower layer. The hydrogel flake was lyophilized for scanning electron microscopy (SEM) analysis in order to evaluate the difference in pore sizes in the internal of the hydrogel. The images revealed that the pores near the upper layer were smaller than those near to the lower layer, which confirmed our hypothesis of the centripetal contraction force (Figure 1o,p,q,n). Similar results (Figure S2, Movie S1, Supporting Information) were also achieved in a finite element analysis in a dual‐layer hydrogel model with different crosslinking density in each layer (a simplified model of the hydrogel with density gradient).

**Figure 1:**
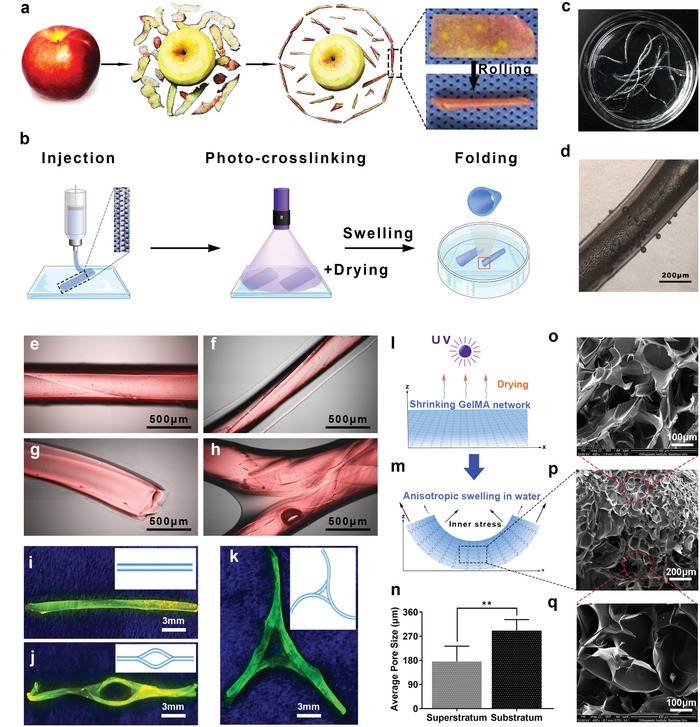
Establishment of the MHT scaffolds. a) The process of drying‐driven curling like “apple peels”. b) The 3‐step forming of the hydrogel‐based MHTs. c,d) Macroscopic and microscopic images of the hydrogel‐based MHTs. The MHTs were stained with rhodamine and excited by a e–h) fluorescent microscopy or i–k) UV light. e,f) Morphology of single MHTs. g) The cross section of an MHT. h) Morphology of a branched MHT. i) Morphology of a single MHT. j) Morphology of an MHT circle. k) Morphology of a T‐shaped MHT. l,m) Scheme of the curling of the hydrogel flake driven by the heterogeneous internal strain after re‐swelling, caused by non‐uniform upper‐lower density after photocrosslinking and drying. p) SEM images of the lyophilized GelMA hydrogel after crosslinking, drying, and swelling. o,q) Detail view of the upper and lower layer in (p). n) Pore size in (o) 176 ± 23 µm and q) 292 ± 17 µm, **p < 0.01 (*n* = 6).

It is worth mentioning that the diameters of the resulting MHTs could be regulated by changing the dimension of the flake and concentration and crosslinking time of the hydrogel (Figures S3–S5, Supporting Information). Therefore, it was demonstrated that this 3D self‐forming technique could be utilized to fabricate vascular scaffolds with various diameters, especially less than 400 µm, which was extremely difficult to achieve with the previous reducing material and 3D printing strategies.^[^ ^8^, ^10^, ^19^ ^]^ This property made the 3D self‐forming technique highly flexible for meeting the demands of different tissues and organs, which implied its great potential for practical use.

Compared with hollow tubules constructed by other methods,^[^ ^10^, ^26^ ^]^ higher continuality and accuracy were achieved in the GelMA MHTs fabricated by 3D‐shape‐morphing at micro‐scale. For example, rhodamine was added into the GelMA‐Irgacure solution during fabrication of the MHTs (Figure 1). The integrity and uniformity of the walls of the tubules, which benefited the migration of the cells,^[^ ^18^ ^]^ were maintained to the greatest extent, since the MHTs were formed by the transformation of an unabridged hydrogel flake. At the same time, the edges of the flakes were sometimes left unclosed, as illustrated by the linear crack on the MHT wall shown in Figure 1e,f. Such cracks could be closed by increasing the extent of curling of the flake. However, the cracks have their special roles, because of the unique function of the cracks to mimic the natural process of vascularization, from leaky to mature. Furthermore, the cracks enabled the migration of cells into the MHTs at the early stage of vessel reconstruction,^[^ ^6^ ^]^ which benefited the sprouting of the neovessels, and were eventually filled by the fresh endothelial cells to form integrate vessels at the late stage of vessel remodeling. Moreover, the morphology of the MHTs could be effortlessly changed by controlling the shape of the original hydrogel flake. For example, a branched flake re‐swelled into a branched vascular scaffold, as shown in Figure 1h. This also expanded the prospect in application of 3D‐shape‐morphing in the construction of vascular scaffolds. For example, vessel connections with different types of anastomosis could be achieved by making different branches in the flakes, such as arteriovenous anastomosis ring (Figure 1j) and Willis arterial circle in the basis encephali (Figure 1k). In summary, the 3D‐shape‐morphing technique is of great flexibility and potential to lay the technique foundation for the construction of complex vascular network which was difficult to be realized directly in traditional ways.

Next, the characteristics of the MHTs were studied on a micro‐scale under SEM after lyophilization. The front face of the tubule was displayed in **Figure** **2**a,e and the broken end of the tubule, are shown in Figure 2b,f. The macro‐morphology of the MHTs remained integrate throughout the lyophilization. Interestingly, the process of 3D‐shape‐morphing resulted in two types of curling on the micro‐scale: 1) as shown in Figure 2a–d, one edge of the flake curled toward the opposite edge around one axis to form an omelet‐shaped pattern; and 2) as shown in Figure 2e–h, two opposite edges curled around two axes independently, resulting in a pattern of biaxial handscroll. Such phenomenon was controlled by the thickness of the center and edge of the flake, as it is shown in Figure 2b,f, where the thickness of the wall of tubule was not uniform. To verify this point theoretically, continuum models of the two curling patterns were established by finite element analysis. The simulations of the rolling of hydrogel tubes were performed in a commercial software ABAQUS 2017 by programming a user material subroutine for swelling of hydrogels,^[^ ^27^ ^]^ and in agreement was found between the simulations (Figure 2c,d,g,h) and experiments (Figure 2a,b,e,f). Our simulations revealed that the distribution of thickness of the initial hydrogel sheets played a crucial role in determining the final morphology of hydrogel tubes. We found that in contrast to the case of rectangular sheets (Figure S2, Supporting Information), the bending curvature varied along the width of the hydrogel sheets with non‐uniform thickness—the less the thickness, the greater was the bending curvature. If we placed more materials at the left side (Figure 2d, Movie S2, Supporting Information), for example, a material with a thickness of 0.08 mm, but fewer materials with a thickness of 0.05 mm at the right side, the original straight bilayer system rolled into a tube with a spiral cross section (“omelet”‐shaped pattern). Furthermore, if we placed more materials in the middle than at the sides, as shown in Figure 2h and Movie S3, Supporting Information, it will eventually roll into a tube with a symmetric spiral cross section (biaxial handscroll). We demonstrated that adjusting the parameters of the local thickness of the flakes ultimately altered the pattern of the MHTs. In summary, single pipelines and serial‐parallel pipes could both be achieved by simply adjusting the parameters in shape‐morphing. This is very meaningful to enrich the construction strategies of vascular scaffolds, since the arteries and veins usually travelled concomitantly in vivo. With the “omelet”‐shaped model, it was even possible to form multi‐layered MHTs via intensified curling of the flake, and independent culture of different cell on the inner and outer walls of the MHTs could make vascular tissues into more complicated structure, such as the three layers of the arteries, tunica intima, tunic media, and tunica adventitia. Taking the branched tubules and vascular circles (as discussed above) together, 3D‐shape‐morphing held a broad perspective in the tissue engineering of complicated and biomimetic vascularization and even neural repair.

**Figure 2:**
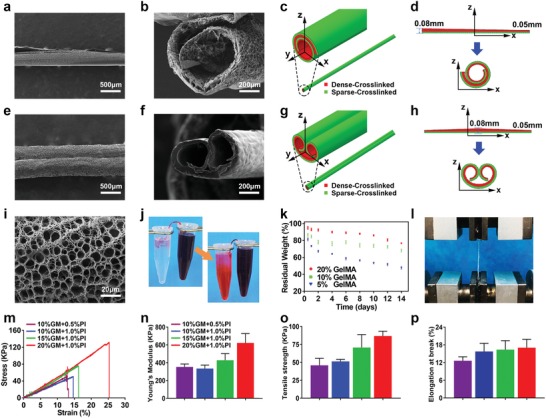
Physical characterization of the MHTs. a,b) SEM image of the “omelet”‐like MHTs. e,f) SEM images of the biaxial handscroll‐like MHTs. c,d,g,h) FEM analysis of the two types of the MHTs. FEM simulations of 3D‐shape‐morphing of hydrogel tubes. The gradient of material properties across the thickness of the hydrogel sheets was simplified by a bi‐layer denoted by “dense‐crosslinked” and “sparse‐crosslinked”, indicated by red and green respectively. Two different morphological types were achieved by controlling the thickness distribution of the materials along the width of the hydrogel sheets. c)“Omelet”‐like hydrogel tube with a spiral cross sectiond) was achieved by placing more materials at the left side than at the right side, g) biaxial handscroll‐like tube with a symmetric spiral cross section h) was achieved by placing more materials in the middle than at the sides. i) Detail view of the mesoporous structure of the wall of the MHT. j) Siphon effect of the MHT. k) Degradation profile of GelMA hydrogel. l) Stress–strain test for MHT. m) Stress–strain curve of MHTs fabricated with different hydrogel concentration. n,o,p) Young's modulus, tensile strength, and stretching capacity of the MHTs, derived from the stress–strain curve.

Moreover, since the walls of the tubulars were composed of crosslinked hydrogels, the mesoporous structure of the walls was observed after lyophilization (Figure 2i). As an important advantage of hydrogels as biomaterials, porosity and water‐permeability promoted the efficacy of the transportation of oxygen, nutrient, and metabolites, as well as the water retention of the material.^[^ ^28^, ^29^ ^]^ While previous studies demonstrated that the swelling ratio of electrospun GelMA fibrous mats could reach 600%,^[^ ^6^ ^]^ the hydrogel flake prepared in our study showed a swelling ratio of ≈400%. Additionally, it was noteworthy that as the flake curled into a MHT, siphon effect was generated by its micro‐channel, which significantly enhanced the water retention of the material, as demonstrated in later sections. Overall, the special micro‐scaled properties of the MHTs created a proper environment for the adhesion, growth, and migration of the cells, which would undoubtedly accelerate the process of the vascular regeneration and remodeling.

The ability to transport fluid or blood with nutrient and metabolites is very important, while investigating the character of MHT. As shown in Figure 2j, driven by the siphon effect, the red ink was transported from one end to the other through the MHT, simulating the in vivo function of the vessels to transport nutrients and metabolites. At the same time, the siphon effect also enhanced the water retention. This feature was very advantageous in the early stage of wound healing and tissue repairing. For example, it assisted the clearance of wound effusion and transportation of nutrients without hampering tissue regeneration with its small volume and stiffness (as demonstrated in our in vivo study).^[^ ^28^, ^30^, ^31^ ^]^

Degradability was an important parameter of biomaterials, which could directly influence the angioplasty and reduce foreign body reaction.^[^ ^32^ ^]^ In vitro degradation of hydrogel made by 20%, 10%, 5%GelMA in 14 days were investigated, as shown in Figure 2k. In the first 2 days, hydrogels made by 10–20%GelMA degraded rapidly to 78–93% of its original weight, possibly due to the release of the uncrosslinked GelMA monomers. The degradation then went through a platform stage, followed by an accelerated process from day 8 or 10, leaving 47%, 68%, and 77% of their original weight at day 14, respectively in hydrogel made by 5%, 10%, 20%GelMA. The critical period of vascularization was also day 3–7, corresponding with the platform stage of the degradation of GelMA. Therefore, after bringing its effects as scaffold to fully play, the MHTs degraded rapidly, which reduced foreign body reaction. Moreover, the difference between groups indicated that the degradation rate of the hydrogel was adjustable, which was of wide application for different tissue environment.^[^ ^33^ ^]^

An ideal 3D tissue engineering scaffold for vascularization should also have good mechanical properties and degradability to enable cell growth and migration, while maintaining the morphology and function of the new tissue in a certain temporal‐spatio structure.^[^ ^34^ ^]^ The mechanical study showed the typical tensile stress–strain curve on the hydrogel‐based MHTs (Figure 2l,m). As shown in Figure 2n–p, the Young's modulus, tensile strength, and maximum length of the MHTs were mainly dependent on the density of the scaffold, that is, the mechanical strength of the MHTs was positively correlated to the concentration and degree of crosslinking of the hydrogel. Vascular surgical research reported the limited stretching capacity of the vessels, for example, vein vessels only had a stretching capacity of 7%.^[^ ^35^ ^]^ Furthermore, our fabricated hydrogel‐based MHTs was able to be stretched to 13–17% of its own length (Figure 2p). Therefore, in theory, the MHTs could fulfill the mechanical demands of vascular scaffolds for all types of tissues. Moreover, the customized scaffold with a specific strength could be fabricated by adjusting the density and thickness of the tubular wall to meet the needs of any typical of vessels.

When it came to the strength of scaffolds, especially the scaffolds for vascularization, the soft and water‐permeable materials showed more advantages, and the hydrogels definitely outperformed the other biomaterials. For example, GelMA hydrogel could absorb water of 6 times of its own weight, while maintaining good permeability. In the process of vascularization, these properties endowed them with extracellular matric, simulating characteristics to transport metabolites, nutrients, and cytokines. In summary, in view of the excellent physical properties, such as mechanical properties, hydrophilicity, degradation, and so forth, hydrogel was the optimized material to construct scaffold to accelerate vascularization.

Biocompatibility is a key point in the construction of tissue engineering scaffold. Given that the 3D‐shape‐morphing hydrogel scaffold went through multiple processes, such as drying during fabrication, CCK‐8 assay was conducted to investigate the metabolic activity of human umbilical vein endothelial cells (HUVECs) seeded on GelMA surfaces after various processing for 1, 3, 5 days (**Figure** **3**f). As indicated from the results, the drying process had no significant influence on the metabolic activity of the HUVECs. It has been shown in previous studies that the photoinitiator in the photocrosslinkable hydrogel system was cytotoxic.^[^ ^4^ ^]^ Therefore, the MHTs were pretreated by soaking in DDW for 2 h to remove the unreacted PI. It was found in the CCK‐8 results that the pretreatment significantly improved the cell viability after 3 days incubation compared to the non‐treatment group. After replacement of the medium with fresh medium, the PI concentration in the non‐pretreatment group slumped, raising their cell viability rapidly to that of the pretreatment group. Interestingly, during the 3D forming of the MHT, the hydrogel flake went through a process of soaking and reswelling, which assembled the process of the pretreatment. Therefore, the 3D forming method was also of great significance to improve the biocompatibility of the scaffolds in the case of PI‐initiated hydrogels.

**Figure 3:**
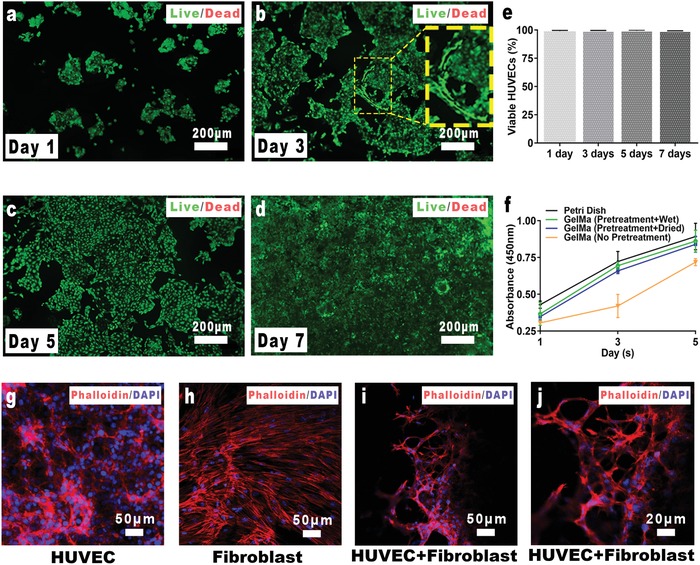
Biocompatibility of the GelMA hydrogel. a–d) Live/dead staining of HUVEC cultured on GelMA for 1, 3, 5, 7 days. Green: live cells. Red: dead cells. Yellow lines in (b): lumen spontaneously formed by the HUVECs. e) Proportion of the live cells. f) CCK‐8 results of the cells cultured on the pretreated (soaking or drying) hydrogels. g–j) Cytoskeleton staining of HUVECs, fibroblasts and coculture of both cells on the hydrogel. Red: cytoskeleton marked by phalloidin. Blue: nucleus marked by DAPI.

The cell viability of HUVECs seeded on the GelMA was further analyzed by live/dead staining. The statistical results indicated that 98–99% HUVECs were viable at all time points, demonstrating the biocompatibility of the pretreated GelMA hydrogel (Figure 3a–e). From 1 to 7 days, HUVECs proliferated rapidly on the hydrogel surface and gradually covered the whole surface. Similarly, when the HUVECs were seeded on the wall of an MHT, a new vessel was created morphologically. In particular, a 3D‐micro‐tubule structure spontaneously formed by HUVECs, as shown in Figure 3b. This phenomenon suggests that vascularization in practice is very complicated, for example, new vessel branched can be derived from the main body of the 3D scaffold. It is understandable from the material point of view that the cell affinity and mesoporous structure of the hydrogels benefit the communication between cells and construction of complex tissues.^[^ ^4^, ^36^ ^]^

Furthermore, the vascularization process was investigated from a multicellular perspective by coculturing of HUVECs and fibroblasts on hydrogels. Fibroblasts promoted the tubule formation of HUVECs (Figure 3g–j), while HUVECs alone grew in a “paving stone”‐like manner, were they tended to grow along with the fibroblast into a network‐like structure when cocultured with fibroblasts. Similar effects were also reported in previous studies on mesenchymal stem cells.^[^ ^4^, ^5^ ^]^ It could be inferred that compared to in vitro environment, the in vivo environment could accelerate the process of vascularization, and the scaffold is a key media and environment to connect multiple types of cells.

The growth of HUVECs on the MHTs was also investigated. The MHTs were soaked into cell suspension after lyophilization and UV sterilization, left settled until the cells were adhered on the inner and outer surface of the MHTs. The MHTs were then incubated in fresh medium for 35 days, followed by fluorescent staining and confocal imaging. **Figure** **4** shows the hollow morphology of the MHTs fully covered by HUVECs. The fluorescent image and statistical results of live/dead staining suggest the high viability of the cells on the MHT scaffold (Figure 4a–d,s). The cell survival rate of 1 day after seeding was 88.91%, possibly due to the absorption of dead cells from digestion to the lyophilized scaffold. The survival rate was ≈98% in the following days of incubation, as expected. Tubules curled in two different manners as discussed previously (Figure 4m–p) and semi‐open tubules, bifurcated tubules (Figure S9, Supporting Information) were also seeded with cells by cell adsorption, all of which resulted in the scaffold‐matched artificial vessel. These results demonstrated that scaffold‐matched artificial pre‐vessel could be obtained by cell adsorption on the MHTs constructed in this study, with a high accuracy of even 50–500 µm in diameter, which is very difficult to achieve with other methods.

**Figure 4:**
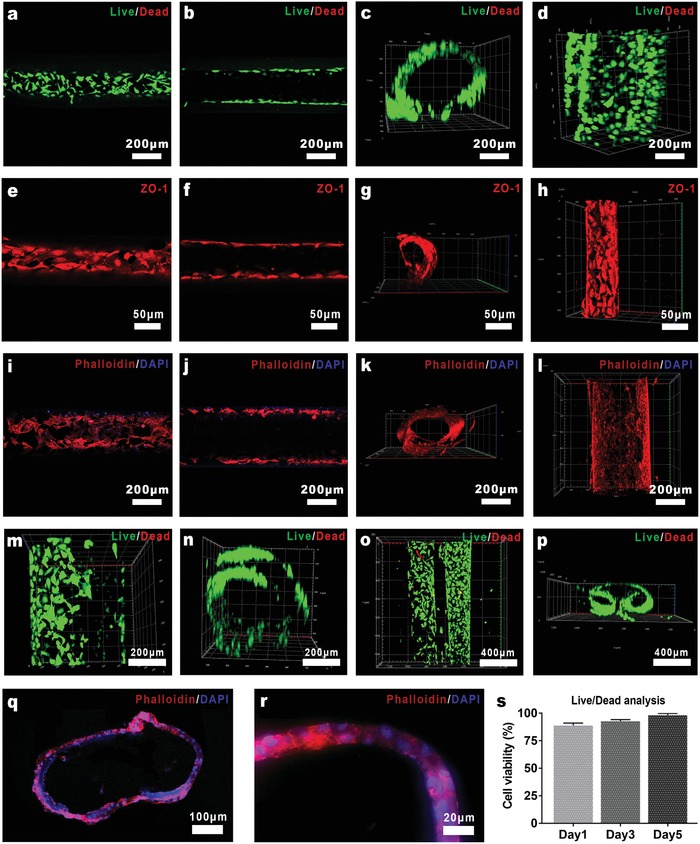
Confocal imaging and 3D reconstruction of the biomimetic vessels developed in vitro. a–d) Survival of the HUVECs incubated for 3 days on the MHTs. s) Proportion of live cells on the MHTs. e–h) ZO‐1 expression on the cytomembranes of HUVECs cultured on the MHTs for 3 days, marked by immunofluorescent staining. i–l) The extension of the HUVECs cultured on the MHTs for 3 days, marked by phalloidin staining. m,n) Live/dead staining of the HUVECs cultured on the omelet‐like MHTs for 3 days. o,p) Live/dead staining of the HUVECs cultured on the biaxial handscroll‐like MHTs for 3 days. q,r) The cross section of the HUVEC seeded MHTs incubated for 3 days, frozen sliced and stained by phalloidin/DAPI.

The growth of HUVECs on the MHTs was further investigated by phalloidin/DAPI staining and confocal imaging. It was observed that on the highly biocompatible MHT scaffold, the intracellular microfilament skeleton of the HUVECs fully extended and communicated with each other, composing an integrate vascular endothelial morphology (Figure 4i–l). The vascularization was not only a simple process of cell growth and migration, but also a process of remodeling and maturation of the vascular structure. Therefore, the HUVEC‐seeded MHTs were frozen‐sectioned after 3 days incubation to obtain a cross section of the biomimetic vessel and stained with phalloidin/DAPI. As shown in the cross section of the lumen in Figure 4q, an integrated and closed lumen structure was composed of the cytoskeleton labeled by red fluorescent, and the cracks on the MHT left by the curling process (as discussed previously) was filled and reconstructed. Meanwhile, connection between cytoskeletons was established by the cells infiltrated into GelMA hydrogel, gradually replacing the original hydrogel tubular wall and transforming into mature vascular endothelial wall (Figure 4r). Furthermore, histologically the period of microvascular remodeling was 3–5 days, which was in accordance with the in vitro vessel construction process with MHTs.^[^ ^5^ ^]^ Taking the GelMA degradation properties together, it was believed that microvessel scaffold constructed with the MHTs perfectly met the requirements of vascularization.

In addition, the function of endothelial cells was gradually perfected during the vascularization. Zonula occludens‐1 (ZO‐1, tight junction markers) played a key role for the endothelium to function.^[^ ^18^ ^]^ The expression of ZO‐1 was detected on the HUVECs of the biomimetic microvessel on 3 days by immunofluorescence. It is shown in Figure 4e–h that there was an abundant expression of ZO‐1 on the cytomembranes, especially between the adjacent cells, suggesting the formation of effective endothelium structure and microenvironment generated by the seeded HUVECs. Especially, in Figure 4h it is shown that the biomimetic microvessel was almost mature at day 3, and kept maturing with the increase of culture time, forming a leak‐free vascular structure composed of intact endothelium.

In the random flap model in rats, necrosis usually occurred at the distal end of the flap due to lack of blood supply.^[^ ^7^, ^37^ ^]^ To validate the MHT‐based microvessel scaffold established in this study, MHTs with diameter from 100–40 µm were implanted under the distal end of the flap, promoting the extension of new vessels from the adjacent area to the distal end of the flap via bridging effect of the scaffold, as illustrated in the Figure 1. From the transformation point of view, this operation converted a random flap into an axial one. It is noteworthy that siphon effect was observed during the operation, which collected the blood leaking from the wound to the area below the flap (**Figure** **5**b), reusing the nutrients from the leaked blood to promote the recruit of cells and new vessels. Necrosis and scabbing occurred on 7 days after operation. The experimental group, implanted with GelMA MHT scaffold, showed a significantly decreased necrosis area of flap (****p < 0.0001) of 21 ± 2% (*n* = 6) compared to the control group (48 ± 1% (*n* = 6)), as shown in Figure 5c1,d1,e. Moreover, differences also emerged in the blood intensity in the necrotic area, as observed by the laser speckle contrast technique results of the flaps. As shown in Figure 5c2,d2,f, the experimental group showed lighter color in the necrotic area with a stronger blood flow signal of 42 ± 2 perfusion units (*n* = 6), showing statistical difference (**p < 0.05) compared to that of the control group (30 ± 1% (*n* = 6)). We can conclude that the GelMA MHT microvessel scaffold is of great importance to improve the blood flow perfusion and survival rate of rat flaps.

**Figure 5:**
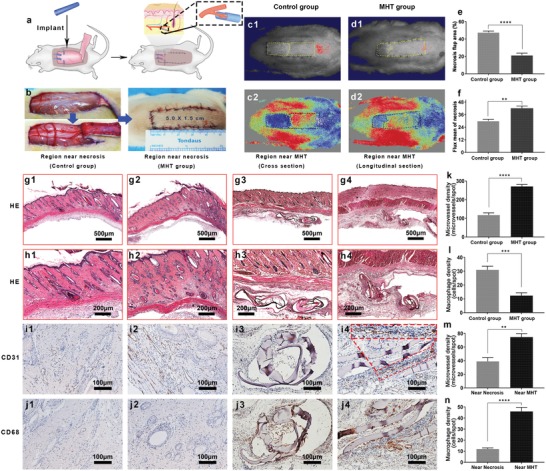
Random flap surgery and analysis on rat dorsal random flap (*n* = 6). a) Scheme of MHTs implantation beneath skin flap. b) Left: immediately and 5 min after placement of GelMA MHTs. Right: in situ suture of the random flap after surgery. c1,d1) necrotic area in the flaps 7 days after surgery of different groups. c2,d2) the laser speckle contrast imaging captured real time blood flow images of different groups. Blood perfusion density increases from blue to red signal. e) Necrotic area proportion in different groups. ****p < 0.0001. f) Average signal intensity of blood flow in different groups. **p < 0.01. g1,g2) H&E staining of the necrosis and survival junction area of different groups skin flaps. g3,g4) H&E staining of region near tube from different slice direction. h1–h4) Detail views of (g1–g4) in high magnification. i1–i4) Immunohistochemical images of groups above showing blood vessel CD31‐positive endothelial cells. Red lines in (i4): a new vessel extending longitudinally into the scaffold. j1–j4) Immunohistochemical images of groups above showing the CD68‐positive macrophage/monocytes. k) average density of the microvessels, ****p < 0.0001, l) average macrophage density of the control and experimental group, ***p < 0.001. m) Comparison of microvessel density in the area near the MHT and necrotic area in the experimental group, **p < 0.01. n) Comparison of inflammation in the area near the MHT and necrotic area in the experimental group, ****p < 0.0001.

HE staining was performed on the necrotic junction of the flaps. As shown in Figure 5g2,h2, little material was observed below the flap, because of the small size of the MHT and the rapid degradation of GelMA. Furthermore, the vascularization near the necrotic area was evaluated. Microvessel in the flaps was marked with CD31 immunohistochemical staining (Figure 5i1,i2) and counted under medium magnification in multiple spots. As shown in Figure 5k, the experiment group exhibited 271 ± 10 microvessels per spot, showing a significant increase (****p < 0.0001) compared to that of the control group (117 ± 12 microvessels per spot). In addition, the tiny area of the residual MHT material was discovered and sliced cross‐sectionally and vertical‐sectionally, as shown in Figure 5g3,g4,h3,h4, and the MVD around the residual material was evaluated (Figure 5i3,i4). It was found out that the neovascularization was concentrated around the material, with some of the vessels extended longitudinally into the scaffold, as marked by red pane in Figure 5i4. The CD31 expression near the MHT area (Figure 5i3,i4) and in the non‐MHT area (Figure 5i2) was counted under high magnification in multiple spots. Superior degree of vascularization was found in the area near the MHT compared to that of the non‐MHT area, with a microvessel count of 75 ± 5 microvessels per spot versus 39 ± 6 microvessels per spot (**p < 0.05), as shown in Figure 5m. In conclusion, the application of MHT effectively improved the microcirculation of the flaps with orientation.

While the MHTs showed excellent performance in vascularization evaluation, the inflammation caused by implantation of the scaffolds, is also important to evaluate. The macrophage density was investigated by CD68 immunohistochemical staining. As shown in Figure 5l, the number of macrophages in the necrotic area of the experimental group (Figure 5j2) was significantly reduced compared to that of the control group (Figure 5j1) (12 ± 2 vs 31 ± 3 macrophage/spot) with a statistical significance (***p < 0.001), from which it was concluded that the scaffold acted positively to reduce the inflammation in the necrotic area of flap. The reduced inflammation in the necrotic area of MHT group verified the effectiveness of MHT. It owed to the vascularization effect of the MHT. With more blood supply in distal area of skin flap, necrosis was reduced with the decreased inflammation. Furthermore, we found that macrophages were more concentrated in the area near the scaffold (46 ± 4 macrophage per spot) compared to the non‐scaffold area (12 ± 1 macrophage per spot), with a statistical significance (****p < 0.0001), as shown in Figure 5j2,j4,n. It was just a limited foreign body reaction. Although a certain degree of inflammation was induced around the implantation spot of the MHTs, large‐scaled inflammation was not raised in the whole flap repair. As result of their small size and tubular diameter, the inflammatory reaction was limited at the implantation site and did not reach the full‐thickness of the subcutaneous tissue. The reduced inflammation caused by implantation of MHTs is crucial when compared to other tissue engineering therapies, such as electrospun membranes and injectable scaffolds.^[^ ^6^, ^7^, ^37^, ^38^ ^]^

Overall, in this study, inspired by the nature curling of “apple peels”, we developed a 3D self‐forming method to fabricate hollow tubules. Based on this principle, extended forms of the hollow tubular scaffolds were achieved, such as “omelet”‐like, biaxial, branched, anastomosis ring, and triangle loop shape, fulfilling the demands of vascular scaffold of all the tissue types. Therefore, this method shows a tremendous potential in future exploitation. Compared to the previous efforts, such as direct 3D printing, the 3D self‐forming method offers a higher accuracy and controllability to fabricate hollow tubules with smaller diameters of 50–600 µm. The cyto‐affinity and biocompatibility of the GelMA‐based MHTs, was demonstrated in vitro. Taking advantage of their excellent physical characteristics, the MHTs were incorporated with HUVECs to construct biomimetic vessels in vitro, confirming their great potential as vascular scaffold. Moreover, the ability of the MHTs to promote vascularization and improve blood supply was validated also in vivo in a random flap model in rats. The developed 3D self‐forming method to construct vascular scaffold is believed to avoid the limitation of bio‐printing of vascular scaffolds, holding a great potential in future applications in tissue engineering vascularization.

## Conflict of Interest

The authors declare no conflict of interest.

## Supporting information

Supporting Information

Supplemental Video 1

Supplemental Video 2

Supplemental Video 3

Supplemental Video 4

Supplemental Video 5
