# Supplementary material for: A Biomimetic 3D‐Self‐Forming Approach for Microvascular Scaffolds
Source: Adv Sci (Weinh). 2020 Mar 1;7(9):1903553. doi: 10.1002/advs.201903553 (PMC7201264; doi:10.1002/advs.201903553)
Supplement: Supplementary file 1 — Supporting Information [file ADVS-7-1903553-s001.pdf]

Copyright WILEY-VCH Verlag GmbH & Co. KGaA, 69469 Weinheim, Germany, 2020.

ADVANCED SCIENCE
Open Access

Supporting Information

for *Adv. Sci.,* DOI: 10.1002/advs.201903553

A Biomimetic 3D-Self-Forming Approach for Microvascular Scaffolds

Liucheng Zhang, Yi Xiang, Hongbo Zhang, Liying Cheng, Xiyuan Mao, Ning An, Lu Zhang, Jinxiong Zhou, Lianfu Deng, Yuguang Zhang, Xiaoming Sun,* Hélder A. Santos,* and Wenguo Cui**

Supporting Information

Biomimetic 3D-self-forming approach for the microvascular scaffolds

Liucheng Zhang, Yi Xiang, Hongbo Zhang, Liying Cheng, Xiyuan Mao, Ning An, Lu Zhang, Jinxiong Zhou, Lianfu Deng, Yuguang Zhang*, Xiaoming Sun*, Hélder A. Santos*, Wenguo Cui*

Dr. L. Zhang, X. Mao, L. Cheng, X. Sun, L. Zhang, and Prof. Y. Zhang
Department of Plastic and Reconstructive Surgery, Shanghai Ninth People's Hospital, Shanghai JiaoTong University School of Medicine, 639 Zhi Zao Ju Road, Shanghai 200011, P. R. China
E-mail: 817073@sh9hospital.org (X. Sun), Zhangyg18@126.com (Y. Zhang)

Dr. Y. Xiang, Prof. L. Deng and Prof. W. Cui
Shanghai Key Laboratory for Prevention and Treatment of Bone and Joint Diseases, Shanghai Institute of Traumatology and Orthopaedics, Ruijin Hospital, Shanghai Jiao Tong University School of Medicine, 197 Ruijin 2nd Road, Shanghai 200025, P. R. China
E-mail: wgcui80@hotmail.com;

Dr. N. An, Prof. J. Zhou
State Key Laboratory for Strength and Vibration of Mechanical Structures, School of Aerospace, Xi'an Jiaotong University, Xi'an 710049, P. R. China.

Prof. H. Zhang
Department of Pharmaceutical Sciences Laboratory and Turku Center for Biotechnology, Åbo Akademi University, Turku FI-20520, Finland

Prof. H. A. Santos,
Drug Research program, Division of Pharmaceutical Chemistry and Technology, Faculty of Pharmacy, University of Helsinki, Helsinki FI-00014, Finland.
And
Helsinki Institute of Life Science (HiLIFE), University of Helsinki, Helsinki FI-00014, Finland.
Email: helder.santos@helsinki.fi

Keywords: Bioinspired material; self-forming; vascular scaffold; biomimetic microvessel

This PDF file includes:

Materials & Methods

Supplementary Figures 1 to 9

Captions for Supplementary Movies 1 to 5.

1. Materials & Methods

1.1 Synthesis of GelMA: GelMA was synthesized as previously described [6].

1.2 Preparation of 3D-shape-morphing MHTs: 10-15wt% GelMA or 1wt% methacrylated hyaluronic acid were chosen for common use and was mixed with 1% photoinitiator (Irgacure 2959, Sigma-Aldrich). Rectangular-shaped GelMA hydrogel flake (approximately 20μL GelMA) with the dimensions of 30 mm×2 mm×0.20 mm was fabricated by 3D printer (CELLINK AB, Sweden) or glass mould. After Crosslinking using 365 nm UV light (YUNHOE, UVPL-4II) for 0.5-3min, the hydrogel slice was fully dried at 25℃, self-rolling phenomenon can be observed. Successfully folded GelMA tubes were immersed in DI water removing redundant photoinitiator for 2h, then freeze-dried for later use. Other forms of GelMA tubes were fabricated following the protocols as described above, except changing the initial structure of GelMA hydrogel flake to branch or circle.

1.3 Characterization of MHTs: Prepared GelMA mixed with 0.3% Rhodamine 123 (Sigma-Aldrich) was used to fabricated 3D-shape-morphing tube. Microscopic fluorescence imagines were acquired by Fluorescence microscope (Zeiss, NY). Macroscopic fluorescence images were taken under UV radiation (λ= 365nm). Freeze-dried tubes were examined using SEM (FEI Quanta 250, the Netherlands).

1.4 Physical characterization of MHTs: The GelMA tubes were shaped from Rectangular-shaped hydrogel with dimensions of 40.00mm × 2.00mm × 0.20mm and freeze-dried. Upon MHT swelling in deionized water, uniaxial tensile tests were performed using Instron 5567 (Norwood, MA) mechanical tester. Initial length of 30 mm between the clamps was reserved. Elongation rate was 2 mm/min. Young's modulus, tensile strength, and elongation at break were calculated from the stress–strain curves.

1.5 Finite element: To simulate the deformation of hydrogel composite sheets, we adopt the theory of thermodynamics of hydrogels and use the continuum finite element method (FEM).[27] The core of the methodology is to construct the free energy density of the hydrogel composites. Adopting the Flory-Rehner model, the free energy per reference volume can be expressed as:

$$W(\boldsymbol{F},C)=\frac{1}{2}NkT\left[F_{iK}F_{iK}-3-2\log\left(\det\boldsymbol{F}\right)\right]-\frac{kT}{v}\left[vC\log\left(1+\frac{1}{vC}\right)+\frac{\chi}{1+vC}\right] \quad (1)$$

where N is the number of polymeric chains per reference volume, kT is the absolute temperature in the unit of energy, $\boldsymbol{F}$ is the deformation gradient tensor, v is the volume of one solvent molecule, C is the concentration of the solvent, and χ is the enthalpy of mixing.

To implement the Flory-Rehner model into finite element method, another free-energy function $\hat{W}(F,\mu)$ can be introduced using a Legendre transformation:

$$\hat{W}(\boldsymbol{F},\mu)=W(\boldsymbol{F},C)-\mu C \quad (2)$$

where μ is the chemical potential prescribed by the environment.

A combination of Equations (1) and (2) gives the desired free-energy function:

$$\frac{\hat{W}(\boldsymbol{F},\mu)}{kT/v}=\frac{1}{2}Nv(I-3-2\log J)-\left[(J-1)\log\frac{J}{J-1}+\frac{\chi}{J}\right]-\frac{\mu}{kT}(J-1) \quad (3)$$

where $I=F_{iK}F_{iK}$ and $J=\det\boldsymbol{F}$ are invariants of the deformation gradient.

Equation (3) can be coded into a user-subroutine for a hyperelastic material (UHYPER) in the finite element package, ABAQUS.

Equation (3) also indicates that this system introduces two independent dimensionless materials parameters: Nv and χ. In our simulations, we will take the values $Nv=0.00167$, which is determined by experimental tests since that NkT represents the shear modulus of dry gel under small-strain conditions; and $\chi=0.1$. When the gel with initial chemical potential μ is submerged in a solvent with environmental chemical potential μ_0, the gel swells (when $\mu_0<\mu$) or shrinks (when $\mu_0>\mu$) until it reach equilibrium state. In our simulations, we fixed the initial chemical potential and tune the environmental chemical potential to simulate the volume change of the active

gel.

In order to study the shape-morphing processes observed in experiments, we simulated hydrogel composite sheets with an identical geometry. The top layer (red) are modeled as linear elastic materials and the Young's Modulus is 0.005 (normalized by kT/v), and Possion's Ratio is 0.49. The bottom layer is considered as environmental sensitive hydrogels (green) simulated by “UHYPER”.

1.6 Degradation of GelMA: Round cross-linked GelMA samples of 1.5cm diameter were prepared for testing degradation as previously described.[6]

1.7 Cell culture: Fibroblasts (ZQ0781, sciencell, USA), Human umbilical vein endothelial cells, HUVECs (HXN180, FDCC, China) were cultured as previously described.[6]

1.8 Cell viability: Cell line crawling slides coated with 10% cross-linked GelMA were prepared as previously described.[6] Live/Dead cell Viability Kit (Life Technologies, NY) was used to test cell viability.

1.9 Cell proliferation on GelMA surface of different treatments: Crosslinked 20uL GelMA in each well of 96-well culture plate was prepared. Different treatments include drying the hydrogel simulating the step in 3D-self-forming, immersing hydrogel in water reducing the redundant photoinitiator. Initial seeded HUVEC concentration was controlled at 10^5 per well. At each time point, CCK-8 assay was conducted and the absorbance intensity was measured (450 nm, Thermo Labsystems, USA). All experiments were repeated thrice.

1.10 Cell seeding on the GelMA MHTs: Initially lyophilized GelMA tubules were sterilized. HUVECs were cultured to 70% confluence in 60mm Petri dish and detached from dish using method of trypsinization. Resuspended to 1mL solution and subjected to 1.5mL sterilized centrifuge tube loaded with prepared GelMA tubules, cell suspension can be absorbed into GelMA structure so that HUVECs attached to the tube wall. After 40 minutes for cell adhesion, the tubules were subjected to 40mm confocal petri dish with DMEM-Complete culturing at standard conditions

(37℃, 5% CO_2) for 3 days. All steps were proceeded with aseptic techniques.

1.11 Frozen section of cell-attached GelMA MHTs: After 3 days of culture, the tubes were fixed in 4% paraformaldehyde for 20 min and immersed in optimum cutting temperature compound, OCT (SAKURA,USA). Frozen samples were cut into slices (Leica CM1520) and stained with phalloidin for microscopic examination.

1.12 Immunofluorescence: Cultured for 3 days, cell-attached GelMA tubes were immunostained with anti-Zonula Occludens-1, ZO-1, (ab96587, Abcam, USA) and Goat Anti-Rabbit IgG (Alexa Fluor® 647) (ab150083, Abcam, USA), followed by Immediate observation using confocal fluorescence microscope (Zeiss, NY).

1.13 Ethical Statements: Male Sprague Dawley rats of 4～6 weeks old were used in the study. Animals were maintained under specific pathogen-free conditions. The animal experiments were in accordance with international ethics guidelines and the National Institutes of Health Guide, concerning the Care and Use of Laboratory Animals.

1.14 Animal model: 5.0 cm × 1.5 cm random flap was created as previously described.[6] Sterilized GelMA tubules absorbing normal saline were placed underneath the distal part of flap. The flap was carefully sutured back to its original position.

1.15 Macroscopic evaluation and histologic analysis: At day 7, SD rats were anaesthetized and the flaps were photographed with a digital camera, the necrosis of the flaps were determined by their color, gross appearance, tissue texture and the moorFLPI blood flow imager using the laser speckle contrast technique to capture real time blood flow images. The length of necrosis area was quantified as the percentage of the flap total length and the results were considered as percentage of skin flap necrosis. 1 cm × 1 cm specimens were harvested from the necrosis and survival junction areas of the flaps for histological assessment. Some areas having residual GelMA tubules were harvested in the same way. All tissue samples were fixed with 4% neutral formalin for 24 h, then the specimens were embedded in paraffin and cross-sectioned into 5 μM slices. The slices were stained

using heatoxylin-eosin for light microscopy.

1.16 Immunohistochemical analysis of CD31, CD68: Immunohistochemiscal analysis was performed as previously described. [6] The sections were observed under microscope. Microvascular density was evaluated at 40× magnification, from the most vascularized area three areas were selected, then the microvessels were counted from those three areas at 100× magnification. The average count of the three areas was considered as the microvascular density. CD68-positive macrophage/monocytes were counted in the same way.

1.17 Statistical analysis: All the experiments were preformed in three replicates. Values are expressed as the means ± SD, and the difference between groups was analyzed by one-way ANOVA with Tukey's and Newman Keuls post tests. Statistical analysis was performed by SPSS (IBM Corp., Armonk, NY, USA). P-value < 0.05 were considered statistically significant difference.

.

2. Supplementary Figures 1 to 9

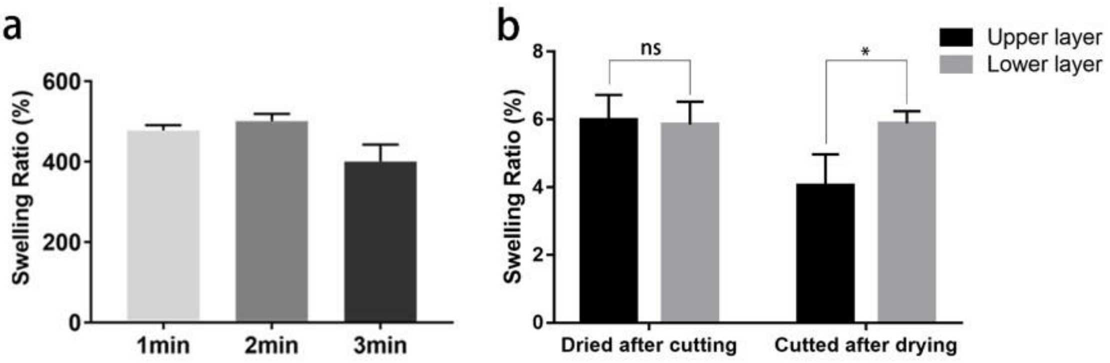

Figure S1: (a) Swelling properties of GelMA hydrogel of different crosslinking time. (b) Swelling properties of GelMA hydrogel of different layers after different treatment. Dried after cutting: hydrogel was cut to upper and lower layers immediately after crosslinking, and then dried; Cut after drying: hydrogel was dried immediately after crosslinking, and then cutted. *P<0.05.

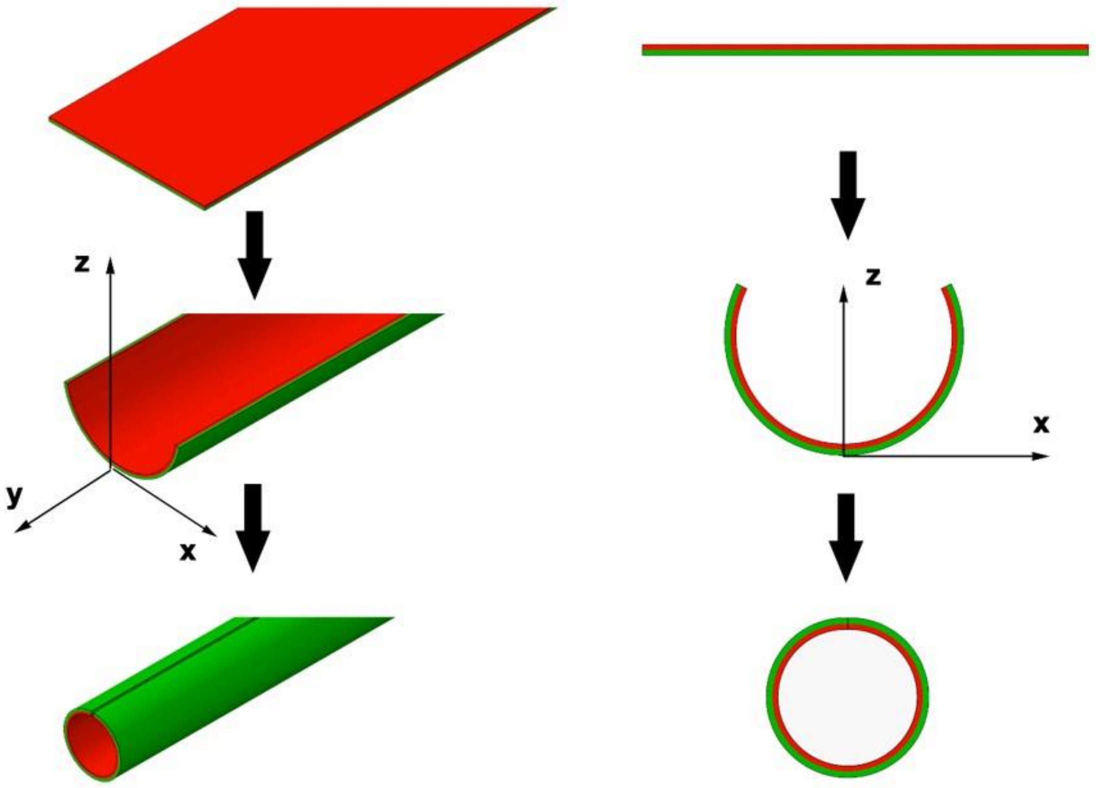

Figure S2: Finite element analysis of the GelMA flake curling into a tubule. Simplified model contained two layers. Red: The densely crosslinked upper layer. Green: The sparsely crosslinked lower layer.

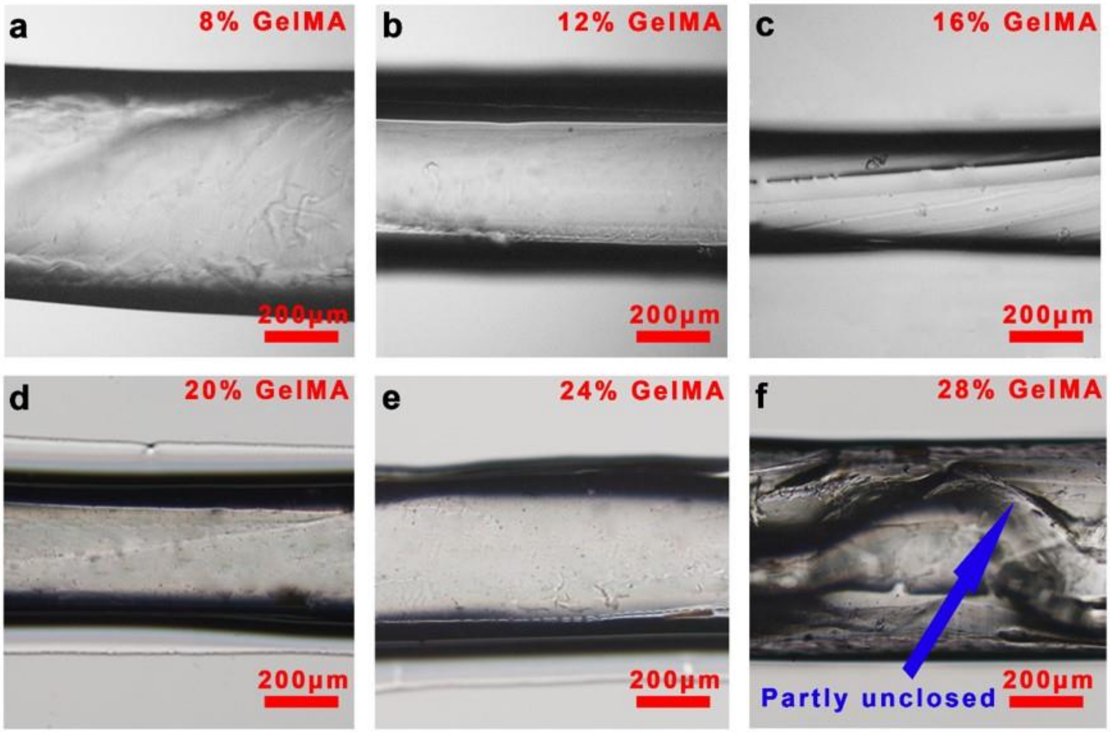

Figure S3: Diameters of the MHTs formed by GelMA of different concentration, bar = 200 μm. Blue arrow: Unclosed part on the tubular wall.

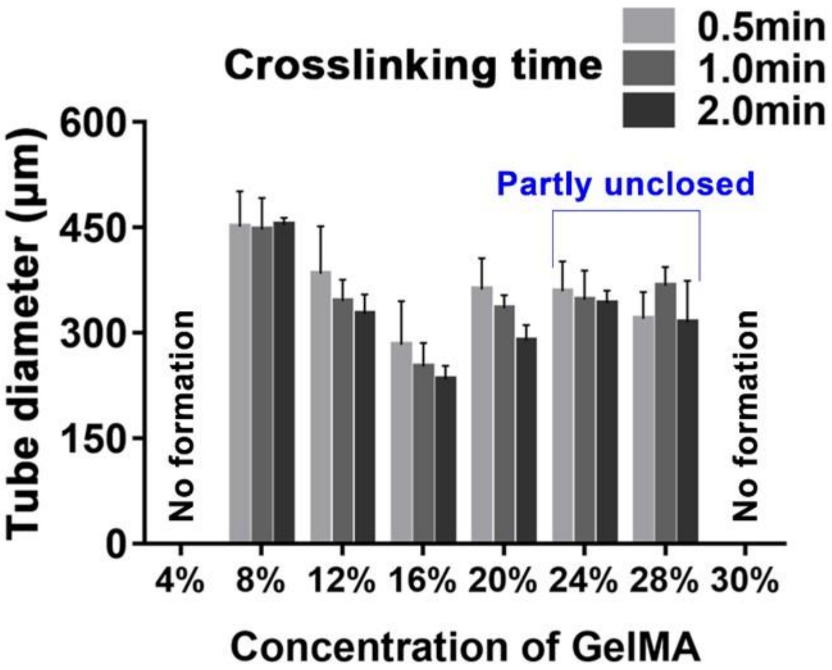

Figure S4: Parameters affecting the 3D-self-forming of the MHTs: GelMA concentration, crosslinking time.

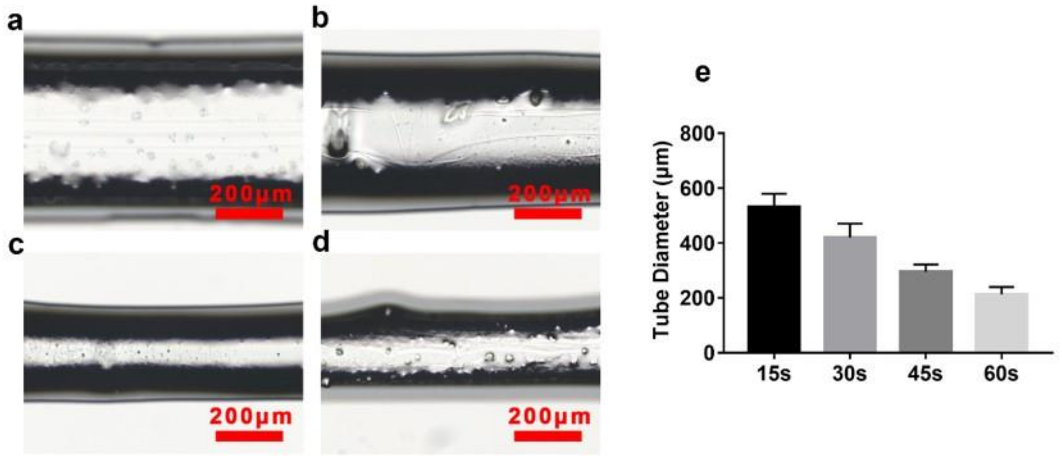

Figure S5: MHTs formed by 1wt% methacrylated hyaluronic acid of different crosslinking time. a-d: crosslinking time 15s, 30s, 45s, 60s, respectively. e: Parameters of MHTs in a-d.

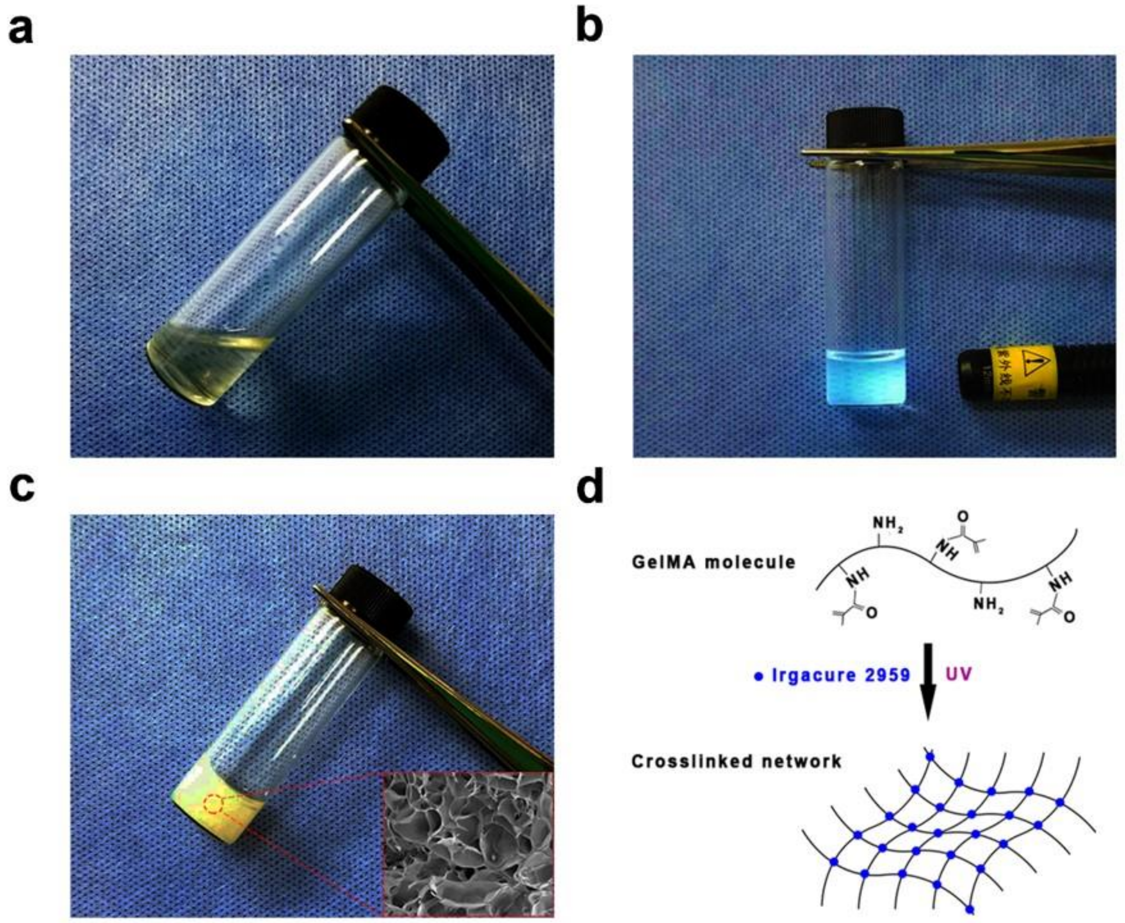

Figure S6: Macroview on photocrosslinking of GelMA. (a) Transparent liquid GelMA pre-gel before crosslinking. (b) UV exposure to initiate the crosslinking of GelMA. (c) The solid GelMA hydrogel after crosslinking, with a reduction of transmittance. (d) Scheme of crosslinking on a molecular level.

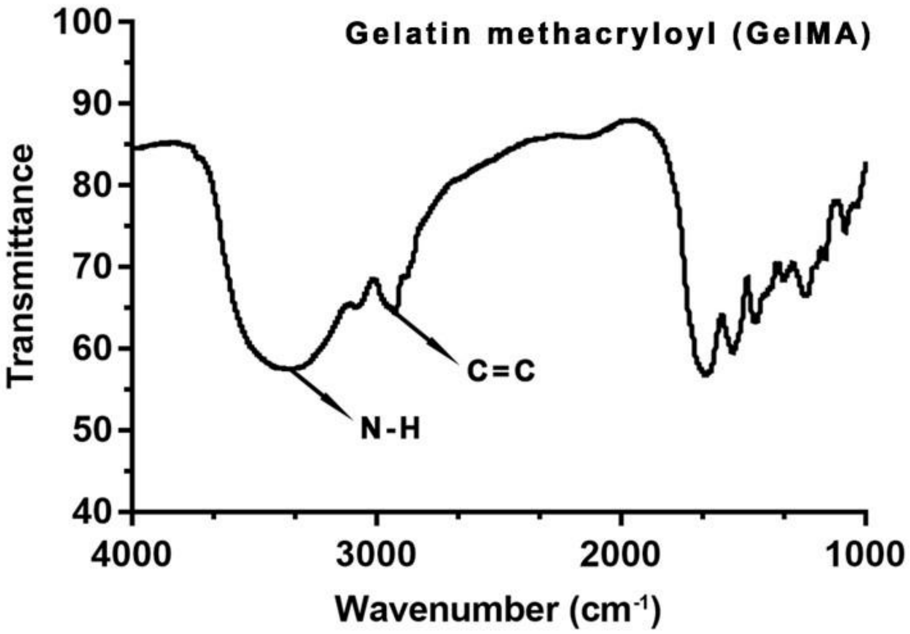

Figure S7: FTIR spectrum of GelMA, C=C representing the absorption peak of the MA groups.

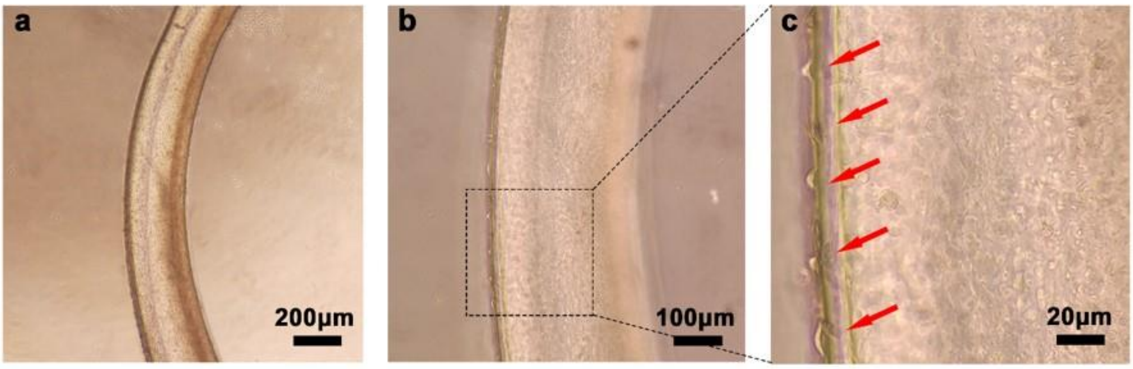

Figure S8: HUVECs adhered on the MHT for 1d. (c) Detail view of the HUVECs adhered in the mesoporous structure of the tubular wall. Red arrow: HUVEC spread on the tubular wall.

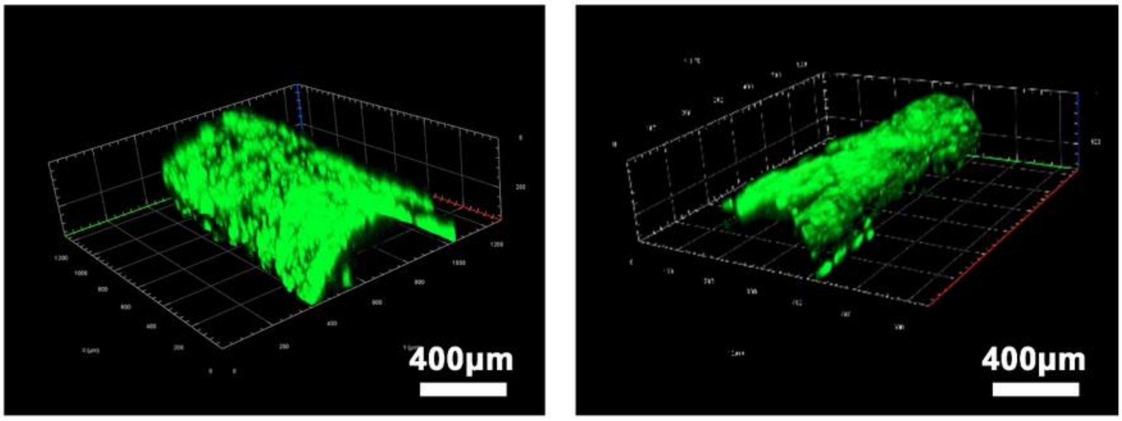

Figure S9: Live/Dead staining of HUVEC seeded on the semi-open MHT. Green: Live cells. Red: Dead cells.

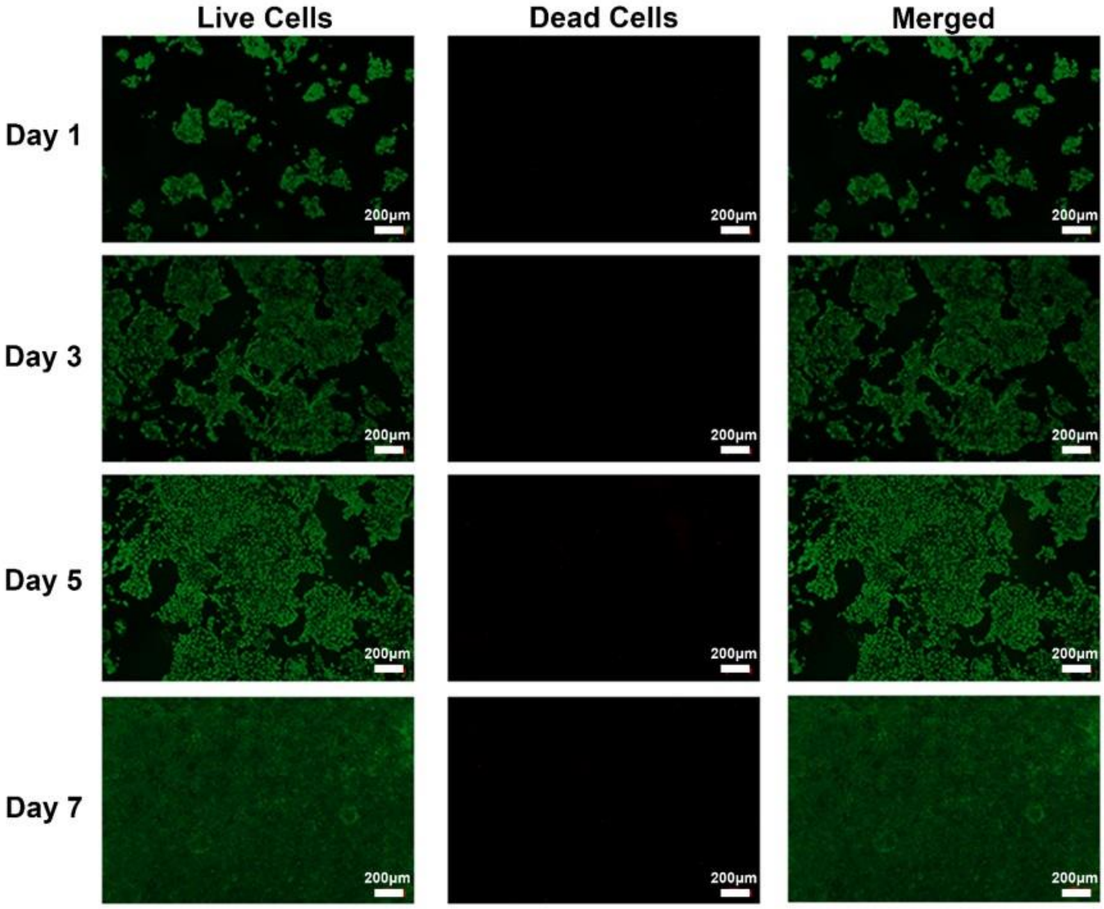

Figure S10: Complete Live/Dead analysis in Figure 3. Green: Live cells. Red: Dead cells.

3. Captions for Supplementary Movies 1 to 5

Supplementary Movie 1: Movie compilation for 3D-self-forming process of MHT. Perfect rectangular-shaped flake with uniform thickness transformed to standard closed tube.

Supplementary Movie 2: Movie compilation for transformation of omelet-like MHT. With more materials at the left side, but fewer materials at the right side, the original straight bilayer system rolled into a tube with a spiral cross-section.

Supplementary Movie 3: Movie compilation for biaxial-handscroll MHT with more materials in the middle than at the sides.

Supplementary Movie 4: Movie compilation showing HUVECs (stained green by Calcein-AM) living on the MHT at day3. This movie supplements Fig. 4.

Supplementary Movie 5: Movie compilation showing morphology of HUVECs on the MHT. Cytoskeleton was stained red by rhodamine-phalloidin. This movie supplements Fig. 4.
